# Characterization of the effects of heat stress on autophagy induction in the pig oocyte

**DOI:** 10.1186/s12958-021-00791-4

**Published:** 2021-07-09

**Authors:** Benjamin J. Hale, Yunsheng Li, Malavika K. Adur, Aileen F. Keating, Lance H. Baumgard, Jason W. Ross

**Affiliations:** Department of Animal Science, Iowa State University, 2356 Kildee Hall, Ames, IA 50011 USA

**Keywords:** Oocyte, Autophagy, Heat stress, Pig

## Abstract

**Background:**

Heat stress (HS) occurs when body heat accumulation exceeds heat dissipation and is associated with swine seasonal infertility. HS contributes to compromised oocyte integrity and reduced embryo development. Autophagy is a potential mechanism for the oocyte to mitigate the detrimental effects of HS by recycling damaged cellular components.

**Methods:**

To characterize the effect of HS on autophagy in oocyte maturation, we utilized an in vitro maturation (IVM) system where oocytes underwent thermal neutral (TN) conditions throughout the entire maturation period (TN/TN), HS conditions during the first half of IVM (HS/TN), or HS conditions during the second half of IVM (TN/HS).

**Results:**

To determine the effect of HS on autophagy induction within the oocyte, we compared the relative abundance and localization of autophagy-related proteins. Heat stress treatment affected the abundance of two well described markers of autophagy induction: autophagy related gene 12 (ATG12) in complex with ATG5 and the cleaved form of microtubule-associated protein 1 light chain 3 beta (LC3B-II). The HS/TN IVM treatment increased the abundance of the ATG12-ATG5 complex and exacerbated the loss of LC3B-II in oocytes. The B-cell lymphoma 2 like 1 protein (BCL2L1) can inhibit autophagy or apoptosis through its interaction with either beclin1 (BECN1) or BCL2 associated X, apoptosis regulator (BAX), respectively. We detected colocalization of BCL2L1 with BAX but not BCL2L1 with BECN1, suggesting that apoptosis is inhibited under the HS/TN treatment but not autophagy. Interestingly, low doses of the autophagy inducer, rapamycin, increased oocyte maturation.

**Conclusions:**

Our results here suggest that HS increases autophagy induction in the oocyte during IVM, and that artificial induction of autophagy increases the maturation rate of oocytes during IVM. These data support autophagy as a potential mechanism activated in the oocyte during HS to recycle damaged cellular components and maintain developmental competence.

**Supplementary Information:**

The online version contains supplementary material available at 10.1186/s12958-021-00791-4.

## Summary sentence

Heat stress induces autophagy in the pig oocyte during oocyte maturation, and autophagy is a potential mechanism by which the oocyte mitigates cellular stress.

## Background

Heat stress (HS) occurs when heat dissipation methods are exceeded by both internal and external heat accumulation [1, 2]. Attempts to maintain euthermia during HS leads to perturbations in physiological processes, such as reduced feed intake, blood flow redistribution to the periphery, and endocrine changes. Collectively these adaptations reduce growth performance, reproductive ability, and alter body composition [3, 4]. These detrimental effects of HS create a financial burden on swine producers and animal agriculture in general. Almost two decades ago, St. Pierre et al. [5] predicted that the combined effects of HS accounted for an approximately $2 billion loss in United States livestock industries. In addition, the economic burden of HS on the United States swine industry alone has been estimated to be $900 million per year [6].

A large portion of the financial burden caused by HS can be explained by a decrease in reproductive efficiency [4]. HS reduces oocyte developmental competence, and induces apoptosis in in vitro fertilized and parthenogenetically activated porcine embryos [7–9]. Oocyte meiotic competence appears to be particularly sensitive to HS, as both pig and bovine oocytes halt meiotic resumption under HS [10, 11]. Furthermore, HS has been shown to impair the gap junctions between oocytes and the surrounding cumulus cells [12, 13]. For oocytes to remain viable, a mechanism is required to maintain homeostasis and mitigate deleterious effects.

Autophagy is a potential stress mitigation response, as it is the process by which damaged cellular components are recycled. There are three major types of autophagy: chaperone-mediated autophagy, microautophagy, and macroautophagy. Macroautophagy (referred to hereafter as autophagy) accounts for the largest amount of cellular resource recycling of the three different types [14]. Autophagy is the sequestration of cytoplasm into a double-membraned cytosolic vesicle, the autophagosome, that fuses with a lysosome to form an autolysosome for degradation by lysosomal hydrolases [15]. The steps of autophagy can be broken down into induction, autophagosome formation, autophagosome-lysosome fusion, and degradation [16]. These processes are marked by the formation of large protein complexes, and much of the regulation occurs post-translationally [17, 18].

During autophagy, Beclin 1 (BECN1) plays a key role in sequestering the nascent lipid membrane that will eventually form the autophagosome [19, 20]. The extension of the autophagosomal membrane around targeted cell debris occurs through two different pathways: One pathway includes the formation of the Autophagy related (ATG)12-ATG5-ATG16 complex, where ATG7 acts like an E1-activating enzyme to conjugate ATG12 to ATG5 [21, 22]. The second ubiquitin-like conjugation pathway results in the cleavage of microtubule-associated proteins 1 light chain 3 alpha/beta (LC3A/B), exposing a glycine residue at the C-terminal end. This process results in the conjugation of LC3 with phosphatidylethanolamine (PE), ultimately forming LC3-II [23], which is a well described marker of mammalian autophagy [24, 25].

Autophagy and apoptosis are regulated in tight coordination, partly through B-cell lymphoma 2 (BCL2) family member proteins, important regulators of apoptosis during mammalian ovary development [26–30]. The dual role of BCL2 family members to regulate both autophagy and apoptosis is mediated by the ability of BCL2 and BCL2 like 1 (BCL2L1; also known as BCL-XL) to prevent apoptosis by inhibiting the formation of mitochondrial pores that release cytochrome C, via interaction with BCL2 associated X, apoptosis regulator (BAX) [31], while BCL2 and BCL2L1 can also interact with BECN1 to regulate autophagy [32, 33].

Both basal and stress-induced autophagy have been observed in the embryo and oocyte. Deficiencies in autophagy-related genes negatively affect both early and late stage embryonic development [34–37] and embryos can also respond to external stressors by inducing autophagy [38, 39]. In the oocyte, autophagy related gene 5 (Atg5) knock-out mice fail to develop past the 4-cell embryonic stage [40]. Furthermore, LC3-II is detectable during initial culture of pig oocytes [41], and BECN1 has been observed in the mouse oocyte [42].

Autophagy represents a potential molecular mechanism by which the oocyte could mitigate the detrimental effects of HS. We have previously utilized an in vivo model to demonstrate that HS affects autophagy-related proteins in the pig ovary, increases the abundance of autophagosome-like structures in follicles, as well as increases BCL2L1 in the ovary [43]. Our working hypothesis is that HS upregulates the autophagy pathway in the oocyte, and thus our study objective was to characterize the induction of autophagy in response to HS during in vitro maturation.

## Methods

### In vitro maturation

Pig ovaries were obtained from a local abattoir for isolation of cumulus-oocyte-complexes (COCs) to be subjected to in vitro maturation (IVM) [44, 45]. Briefly, follicles (2–4 mm) were aspirated and COCs were collected and washed in TL-Hepes with 0.1% polyvinyl alcohol (PVA). Cumulus oocyte complexes were cultured in maturation media (Tissue Culture Media 199 (TCM-199)) containing 0.57 mM L-cysteine, follicle stimulating hormone (0.5 μg/mL), luteinizing hormone (0.5 μg/mL), and epidermal growth factor (10 ng/mL) for approximately 42 h at 38.5 °C in 5% CO_2_. Prior to IVM, a representative sample of germinal vesicle-intact (GV) stage oocytes for each replication were randomly selected from the COC pool. Identification of polar body was performed with phase-contrast light microscopy. Oocytes used for analysis were stripped of cumulus cells via gentle vortex (6 to 8 min) in 1% hyaluronidase in TL-Hepes-PVA and washed in TL-Hepes-PVA. To observe the effect of HS on IVM of oocytes, oocytes underwent three different IVM temperature treatments: 1) TN conditions (38.5 °C) for the entirety of 42 h (TN/TN), 2) TN conditions for the first 21 h of IVM followed by HS (41 °C) conditions for the following 21 h (TN/HS), or 3) HS conditions for the first 21 h of IVM followed by TN conditions for the following 21 h (HS/TN). Following IVM oocytes were stripped of cumulus cells by vortexing 5–6 min in TL-Hepes-PVA supplemented with 1% hyaluronidase and washed in TL-Hepes-PVA.

To characterize the temporal change in abundance of LC3B-II, oocytes were matured under either the TN/TN or HS/TN treatments. A pool of oocytes was collected before introduction into maturation media (0-h). Oocytes were collected after 21 h of either TN (21-h TN) or HS conditions (21-h HS). The remainder of the oocytes that had experienced 21 h of TN conditions were allowed to continue maturation under TN conditions for a total of 42 h (42-h TN/TN), and the remainder of the oocytes that had experienced 21 h of HS conditions continued maturation under a subsequent 21 h of TN conditions, for a total of 42 h (42-h HS/TN).

### Western blot analysis

Pools of 50 denuded oocytes per replicate were collected as described above after 21 or 42 h of IVM. Oocyte pools were lysed in 5 μL of Laemmli sodium dodecyl sulfate buffer at 95 °C for 4 min followed by 1 min on ice and then centrifugation at 1000 rpm for 1 min at room temperature. Lysates from fifty oocytes were loaded per lane of a 4–20% Tris glycine gel (Lonza PAGEr Gold Precast Gels). The BioRad Mini PROTEAN Tetra System was used to separate protein homogenates at 60 V for 30 min followed by 120 V for 90 min. The protein was transferred to a nitrocellulose membrane for 1 h at 100 V at 4 °C. Membrane blocking was conducted using 5% milk in phosphate buffered solution with 0.5% Tween 20 (PBST) for 1 h at room temperature. A rabbit anti-BECN1 (Cell Signaling Technology, 3495), rabbit anti-LC3B (Cell Signaling Technology, 3868), rabbit anti-ATG12 (Cell Signaling Technology, 4181), rabbit anti-BCL2L1 (Cell Signaling Technology, 2764), or normal rabbit IgG (Cell Signaling Technology, 2729) as a negative control were added (1:1000 dilution) to the membrane in 0.5% milk in PBS overnight at 4 °C. Following primary antibody incubation, the membranes were washed with PBST (PBS with 0.1% Tween) three times at room temperature for 10 min each. Donkey anti-Rabbit IgG (Amersham ECL NA934) was incubated (1:1000) with the membrane for 1 h at room temperature. The membrane was then washed three times for 10 min each at room temperature. Horseradish peroxidase substrate (Millipore, Billerica, MA) was added to the membrane for 1 min in the dark, and was exposed to x-ray film and developed for visualization. Average pixel intensity for the band corresponding to the primary antibody was compared for each blot using Image Studio Lite (Li-Core). Signal from detection of each protein of interest was normalized to α-tubulin.

### Rapamycin oocyte IVM and activation

Cumulus-oocyte-complexes were collected and subjected to IVM under the HS/TN treatment as mentioned above, with the addition of either DMSO vehicle control, 1.0 nM rapamycin, 10 nM rapamycin, or 100 nM rapamycin. In addition to rapamycin, IVM media for this study contained 10 ng/mL leukemia inhibitory factor (LIF; Sigma-Aldrich L5283), 40 ng/mL basic fibroblast growth factor (Sigma-Aldrich F0291), and 20 ng/mL insulin-like growth factor 1 (Sigma-Aldrich I36769). After 21 h of HS IVM followed by a subsequent 21 h of TN IVM, oocytes were denuded and healthy metaphase II oocytes containing extruded polar bodies were counted as a percent of total oocytes per treatment. Pools of 50 metaphase II arrested oocytes were then flash frozen in liquid nitrogen to be used for downstream analysis.

### Oocyte fixation and immunohistochemistry

Oocytes were collected and denuded, as described above, from different time points during IVM and then fixed and mounted to slides as previously described [46, 47]. Briefly, oocytes were fixed in 4% paraformaldehyde in PBS overnight at 4 °C, and then transferred to 70% ethanol in PBS at 4 °C. Oocytes were permeabilized in 0.5% Triton X-100**™** (Sigma-Aldrich, St. Louis, MO) for 30 min at room temperature. Next, oocytes were blocked in 5% bovine serum albumin (Sigma-Aldrich, St. Louis, MO) for 45 min at room temperature, then incubated with primary antibody overnight at 4 °C. After approximately 24 h, oocytes were washed twice in PBS for 30 min, and incubated with secondary antibody for 1 h at room temperature. The oocytes were then washed twice in PBS for 30 min, and mounted to microscope slides using SlowFade Gold Mountant containing DAPI (S36939, ThermoFisher Scientific, Pittsburgh, Pennsylvania).

### Colocalization of autophagy markers

To label proteins of interest for colocalization, rabbit anti-BCL2L1 (Cell Signaling Technology, 2764S), rabbit anti-BECN1 (Cell Signaling Technology, 3495S), mouse anti-BCL2L1 (Novus**™**, 46,569), and mouse anti-BAX (Novus, 28,566) primary antibodies were used at a 1:200 dilution. Secondary antibodies used were anti-mouse IgG AlexaFluor 647 (Cell Signaling Technology, 4410S) and anti-rabbit IgG FITC (Life Technologies, F2765) at a 1:250 dilution. To control for nonspecific binding of primary antibodies, a pool of oocytes was incubated in normal rabbit IgG (Cell Signaling Technology, 2729) and normal mouse IgG (Cell Signaling Technology, 5415) at a 1:200 dilution instead of primary antibodies. A Leica SP5X MP confocal microscope (Exton, PA) was used to image antibody labeled proteins of interest.

Primary antibodies derived from different species were used so that secondary antibodies with different fluorophores would recognize and bind to the separate primary antibodies within the same fixed oocyte. This made it possible to view two different fluorophores conjugated to secondary antibodies at different excitation wavelengths. 30 oocytes per temperature treatment per time point were visually assessed using a confocal microscope. Three oocytes per temperature treatment per time point of IVM were used in colocalization analysis on sequential focal planes (step size of 1 μm) measuring spatial fluorescence over approximately 50 μm of each oocyte. Fluorescence of DAPI stained chromatin, labeled rabbit anti-BCL2L1 and mouse anti-BAX, or labeled rabbit anti-BECN1 and mouse anti-BCL2L1 was measured.

### Statistical analysis

Statistical analysis of maturation rate, western blot data, and co-fluorescence data was conducted using PROC MIXED in SAS Enterprise Miner Workstation version 14.1 (Carry, NC), where a standard student t-test was used to compare statistical differences. Statistical significance was determined when *P* values were less than or equal to 0.05. The PDC colocalization plugin [48] in the ImageJ processing program [49] was used to calculate Pearson’s and Spearman’s correlation coefficient and scatter plots representing colocalization of signal intensity collected from confocal microscopy of individual oocytes. Corresponding z-stacks with blocks of pixels randomly scrambled were used to determine that the probability of the measured value for Pearson’s or Spearman’s correlation between two color channels was significantly greater than would be calculated if there was only random overlap of the same information.

## Results

### Heat stress decreases oocyte maturation rate

To characterize the effect of HS on maturation, oocytes underwent IVM during one of three temperature treatments: TN conditions throughout the entire 42-h maturation period (TN/TN), HS conditions during the second half of IVM (TN/HS), or HS conditions during the first half of IVM (HS/TN). Oocytes were subject to 21-h HS intervals because we have previously observed that 42 h of HS greatly decreases oocyte maturation and subsequent embryo development [50]. This also allowed us to compare maturation rate of different temperature treatments with downstream protein abundance and localization. Both HS treatments decreased maturation compared to TN/TN control, where the maturation rate of TN/TN oocytes was 66.9 ± 5.0%, the maturation rate of TN/HS oocytes was decreased to 44.9% ± 5.0% (*P* < 0.01), and the maturation rate of HS/TN oocytes was decreased to 33.2 ± 1.6% (*P* < 0.01; Table 1). The maturation rate of HS/TN oocytes decreased compared to the maturation rate of TN/HS (*P* ≤ 0.05; Table 1). These are comparable to previous results from our group [50].

**Table 1:**
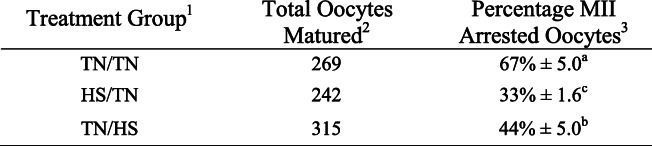
Oocyte maturation to MII arrest under different temperature treatments

^1^Temperature treatment oocytes experienced during IVM: TN throughout the entire IVM (TN/TN) or HS during the first half (HS/TN) or second half (TN/HS) of IVM.

^2^Total number of GV oocytes matured for each treatment from four replicates.

^3^Percentage of MII arrested oocytes from each treatment. Mean ± SEM.

^a,b,c^Values with different superscripts in the same column are significantly different (*P* < 0.05).

### Heat stress affects autophagy-related proteins

**Fig. 1:**
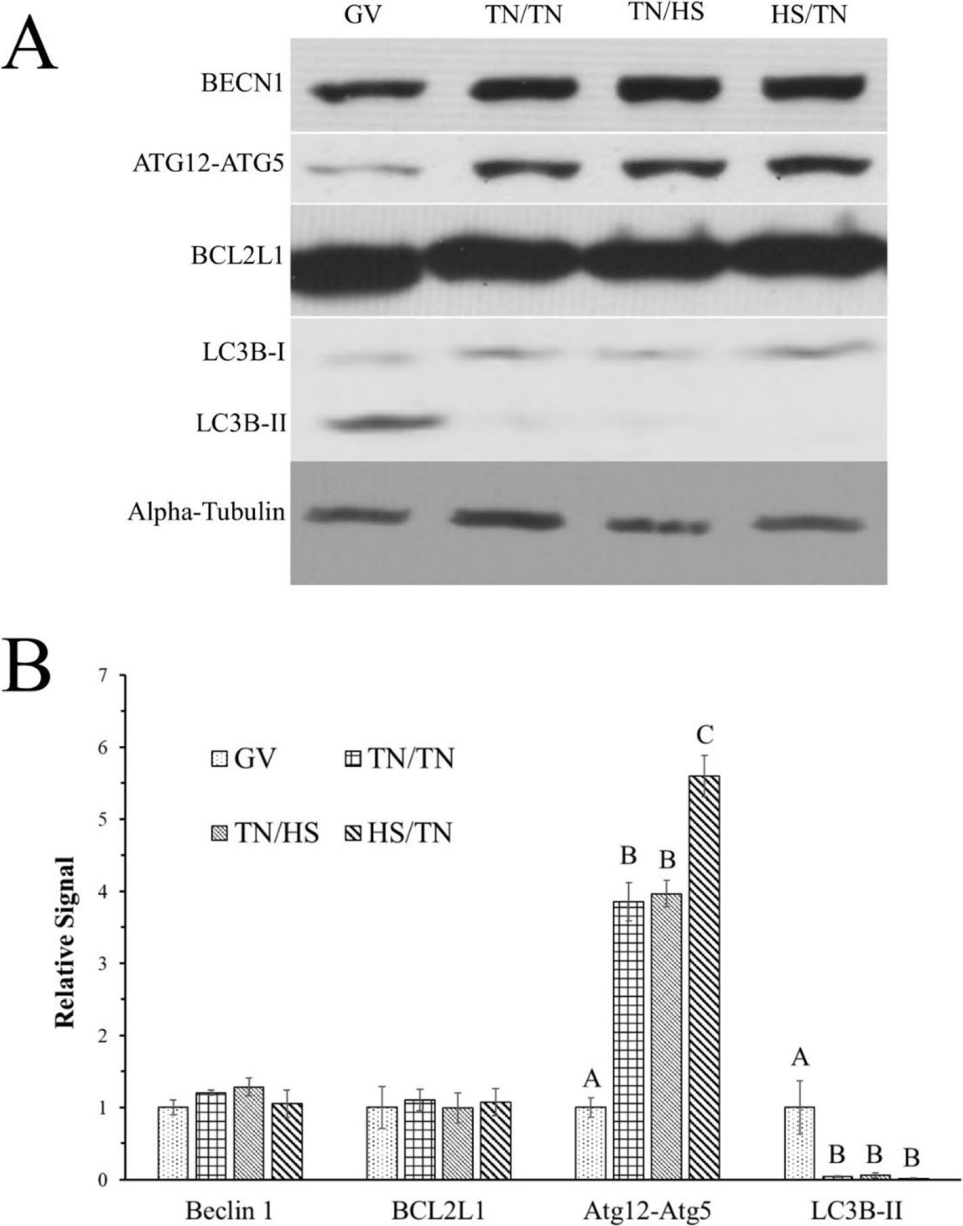
Heat stress during the first half of IVM increases the abundance of ATG12 in complex with ATG5. Oocytes collected from aspirated pig follicular fluid underwent in vitro maturation (IVM) in either TN conditions throughout the entire 42-h maturation period (TN/TN), HS conditions during the second half of IVM (TN/HS), or HS conditions during the first half of IVM (HS/TN). Representative western blots for BECN1, ATG12-ATG5 complex, BCL2L1, LC3B, and α-tubulin (A) from oocytes collected before IVM (GV) and after 42 h of IVM. There was increased abundance of ATG12 in complex with ATG5 after 42 h of IVM under the HS/TN temperature treatment compared to the TN/TN and TN/HS (**B**). Different superscripts within the same protein denotes significant difference (*P* < 0.05)

The abundance of BECN1, fomation of the ATG12-ATG5 complex, and the cleavage of LC3B was measured via western blotting to characterize the effect of HS on autophagy induction in the oocyte. The abundance of BECN1 and BCL2 like 1 (BCL2L1) were unaffected by temperature treatment (Fig. 1A and B). The abundance of ATG12 in complex with ATG5 was increased in oocytes collected after 42 h of IVM of each temperature treatment (TN/TN, TN/HS, or HS/TN) compared to GV-intact oocytes collected before IVM. There was increased abundance (~ 1.5 fold; *P* < 0.01) of ATG12 in complex with ATG5 after 42 h of IVM under the HS/TN temperature treatment compared to the TN/TN (*P* < 0.01) and TN/HS (*P* < 0.01; Fig. 1A and B).

There was no effect of temperature treatment on the abundance of LC3B II in oocytes after IVM with any of the three temperature treatments (*P* > 0.05). Although, there was decreased LC3B-II in TN/TN (*P* < 0.01), TN/HS (*P* < 0.01) and HS/TN oocytes (*P* < 0.01) compared to oocytes collected at 0 h of IVM (Fig. 1A and B). Compared to oocytes collected at 0-h, there was an approximately 24-fold lower abundance of LC3B-II in oocytes collected after TN/TN IVM, approximately 17-fold lower abundance in oocytes collected after TN/HS IVM, and approximately 73-fold lower abundance of LC3B-II in oocytes collected after HS/TN IVM (Fig. 1A and B).

**Fig. 2:**
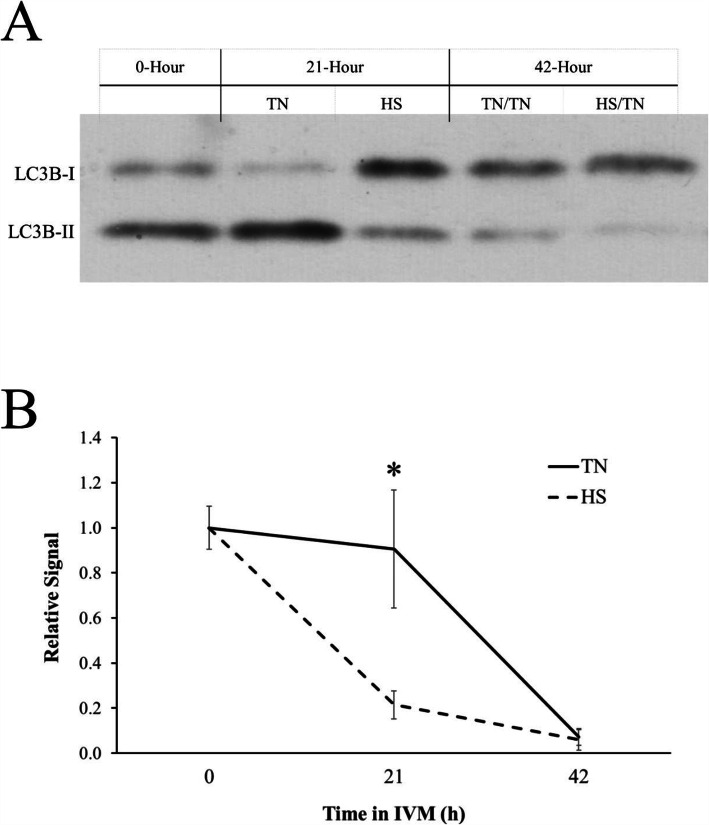
Heat stress exacerbates the decrease of LC3B-II in the oocyte. Oocytes collected from aspirated pig follicular fluid underwent in vitro maturation (IVM) in either TN conditions throughout the entire 42 h maturation period or HS conditions during the first half of IVM (HS/TN). Representative western blot for LC3B from oocytes having undergone TN or HS conditions in IVM after 21 or 42 h or collected before IVM (**A**). There was decreased abundance of cleaved LC3B-II at 21 h of HS IVM compared with 21 h of TN IVM (**B**). Asterisks denotes significant difference (*P* < 0.05) from control

The observed sharp decrease in LC3B-II due to temperature treatment provided rationale for an IVM experiment in which oocytes were collected after 21 h to determine the temporal effects of HS on LC3B-II. Although the abundance of LC3B-II did not differ between TN/TN oocytes and HS/TN oocytes at 42 h of IVM (*P* = 0.92), there was decreased abundance of LC3B-II after 21 h of HS (0.21 ± 0.6 relative band intensity) compared to oocytes collected after 21 h of TN (0.9 ± 0.26; *P* < 0.01; Fig. 2A and B). These data suggest that HS affects the utilization of autophagy-related proteins in the oocyte.

### BCL2L1, BAX, and BECN1 interactions

**Fig. 3:**
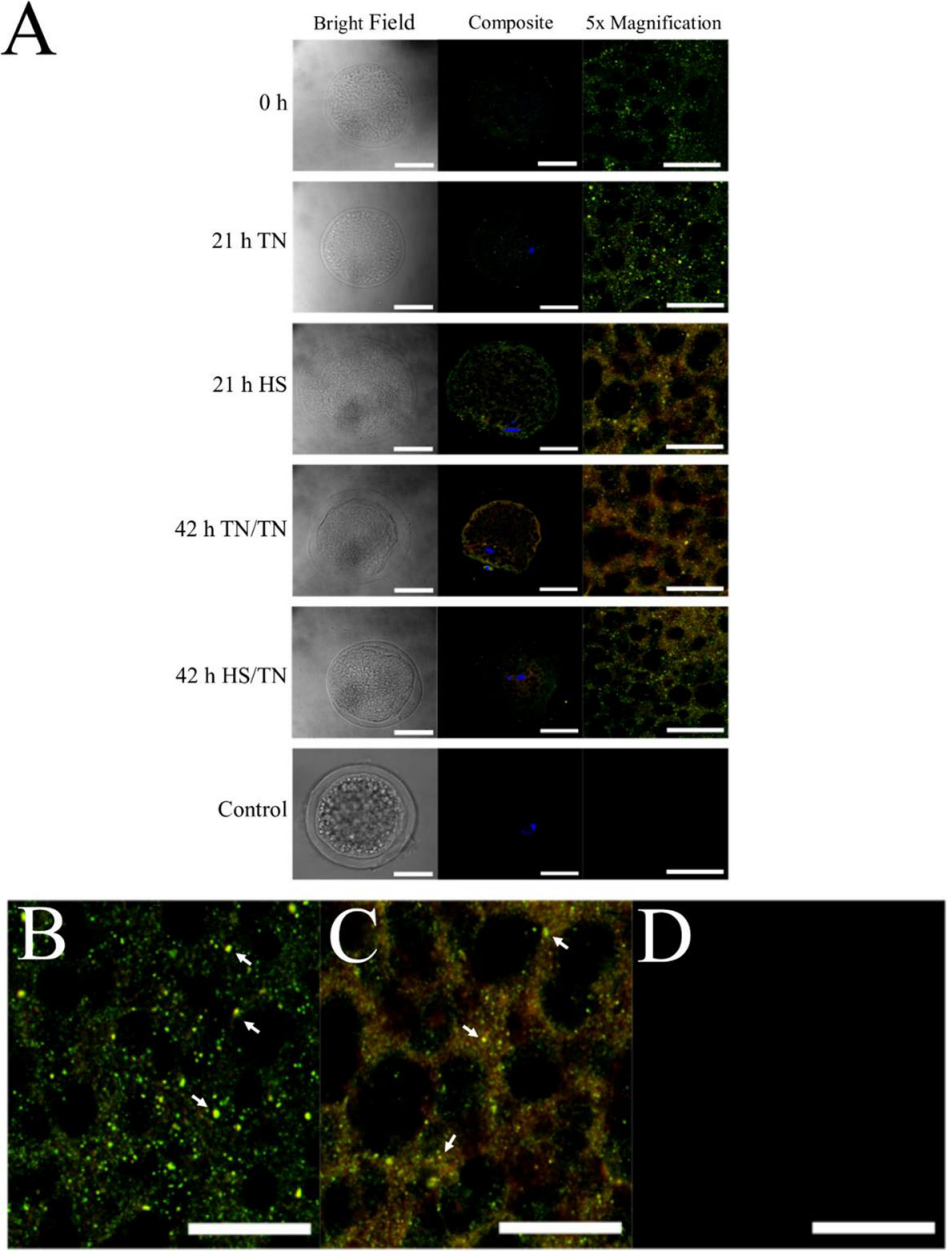
Colocalization of BAX and BCL2L1. Oocytes that underwent either TN/TN IVM or HS/TN IVM were fixed and used for IHC to determine colocalization. Neither the time point during IVM nor the temperature treatment affected colocalization of antibody-labeled BCL2L1 and BAX in the oocyte. Based on level of fluorescence, there appeared to be a qualitative increase in BAX (red) at 21 h of IVM under HS compared to 21 h of IVM at TN conditions, though there appeared to be no effect on the abundance of BCL2L1 (green; **A**). The comparison between 21-h TN (**B**), 21-h HS (**C**), or negative control (**D**) shows that there was punctate yellow signal representing colocalization in both TN and HS oocytes. For **A**, the white bar in images in the left and middle columns represent 50 μm, and the white bar in images in the right column represents 20 μm. The white arrows point to areas of colocalization. The images in the right column are magnified 5× images from the same oocyte from the middle column

Because HS in the first half of IVM sharply decreased oocyte LC3B-II, the interaction of BCL2L1 with either BAX or BECN1 was determined to assess a potential HS-induced regulatory mechanism. The TN/HS treatment was not used in further experiments because it appeared to have a lesser affect on LC3B-II turnover. Oocytes that underwent IVM in either TN/TN or HS/TN conditions were fixed for immunohistochemistry (IHC) to determine BCL2L1, BECN1, or BAX co-localization. Based on the Pearson’s (r_P_) or Spearman’s (r_S_) correlation coefficients, where a r_P_ or r_S_ closer to 1 is indicative of co-localization (Table 2) and overlap of fluorescent signal (Fig. 3A and B), neither the time point nor the temperature treatment affected colocalization of BCL2L1 and BAX. Based on level of fluorescence, BAX was numerically increased, but not significantly increased, at 21 h of HS compared to TN conditions (Fig. S2B). There were no effects of time or temperature treatment on the abundance of BCL2L1 based on level of fluorescence (Fig. S2C).

**Table 2:**
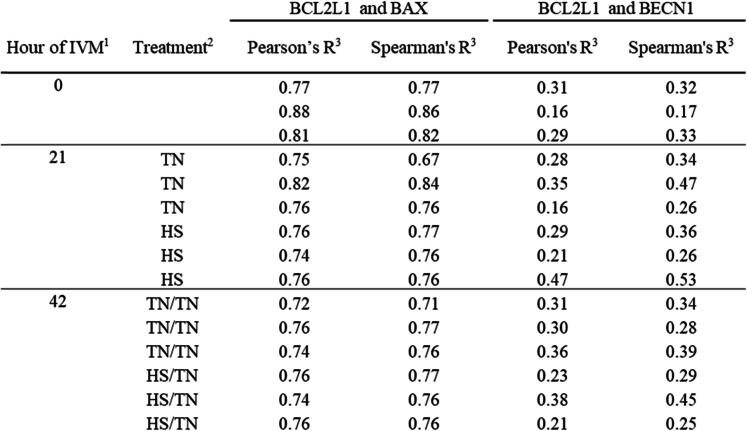
Correlation coefficients indicative of colocalizations

^1^Pools of at least 30 oocytes were collected after 0, 21, or 42 hours of IVM (HS/TN).

^2^Oocytes experienced either TN throughout the entire IVM or HS during the first half of IVM (HS/TN).

^3^The PDC localization plugin in the Image J processing program was used to calculate Pearson’s and Spearman’s correlation coefficient representing colocalization of BCL2L1 with BAX or BECN1 in indivual oocytes. As per French et al. [48], a Pearson’s R of 0. 72 and Spearman’s R of 0.63 is considered a high degree of colocalization.

**Fig. 4:**
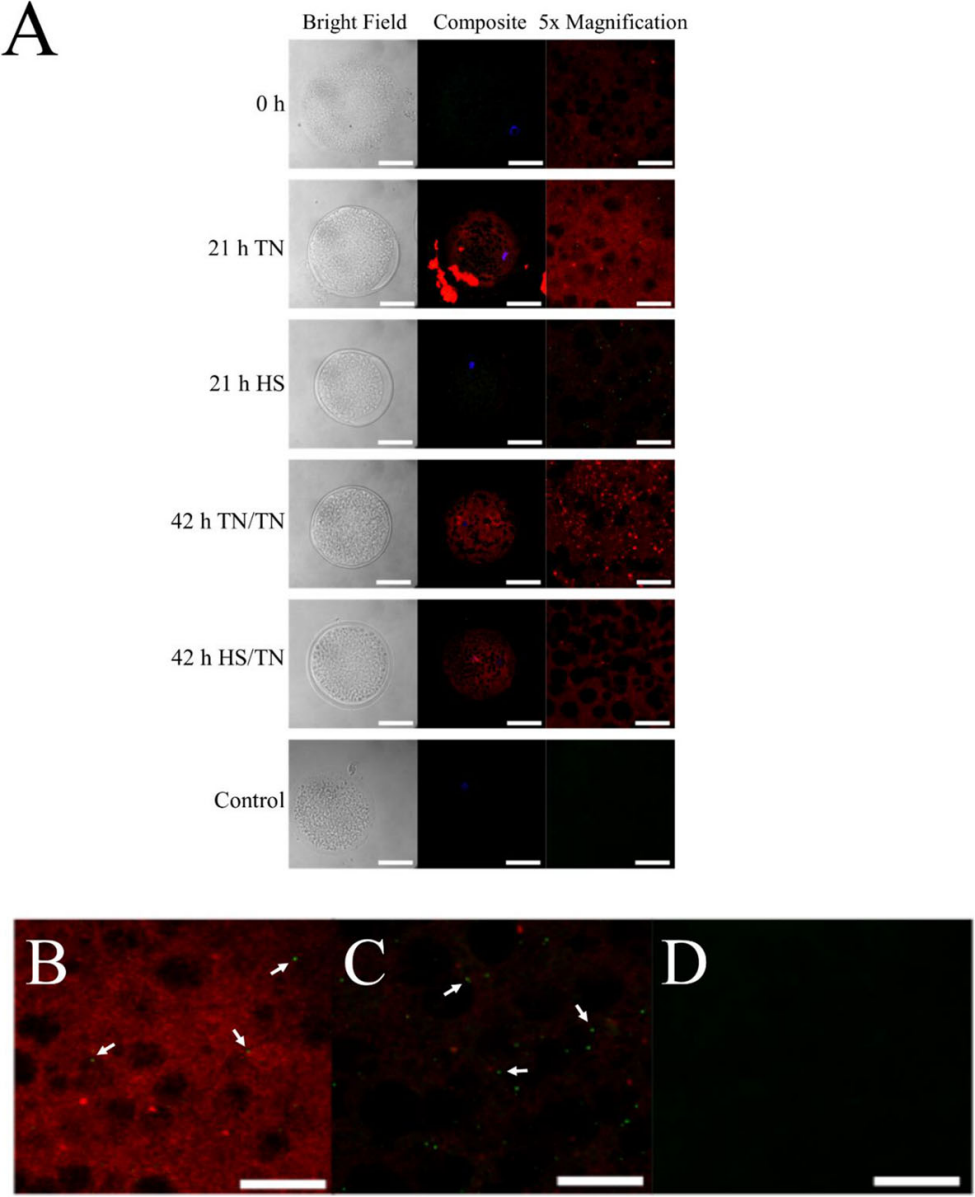
Colocalization of BECN1 and BCL2L1. Oocytes that underwent either TN/TN IVM or HS/TN IVM were fixed and used for IHC to determine co-localization. Colocalization of BECN1 (green) and BCL2L1 (red) was low regardless of time point during IVM or temperature treatment. There appeared to be more punctate intense spots of BECN1 staining in oocytes having undergone 21 h of IVM under HS compared to the oocytes that underwent 21 h of IVM at TN conditions (**A**). The comparison between 21-h TN (**B**), 21-h HS (**C**), or negative control (**D**) shows that there was punctate green signal representing BECN1 but no colocalization in both TN and HS oocytes. For **A**, the white bar in images in the left and middle columns represent 50 μm, and the white bar in images in the right column represents 20 μm. The white arrows point to areas of punctate BECN1 signal but no colocalization. The images in the right column are magnified 5× images from the same oocyte from the middle column

Based on the r_P_ and r_S_ correlation coefficients (Table 2) and overlap of fluorescent signal (Fig. 4A and B), there was little colocalization of anti-BECN1 and anti-BCL2L1 antibodies regardless of time point during IVM or temperature treatment (Table 2). BECN1 staining appeared punctate at all time points and temperature treatment with no overlap with BCL2L1 staining (Fig. 4A and B). There appeared to be more intense punctate BECN1 focal staining in oocytes (Fig. 4A and B). This data suggests that BCL2L1 is participating in the regulation of apoptosis in IVM oocytes under TN or HS conditions, while not appearing to inhibit autophagy.

### Low concentration of rapamycin increases maturation rate

**Fig. 5:**
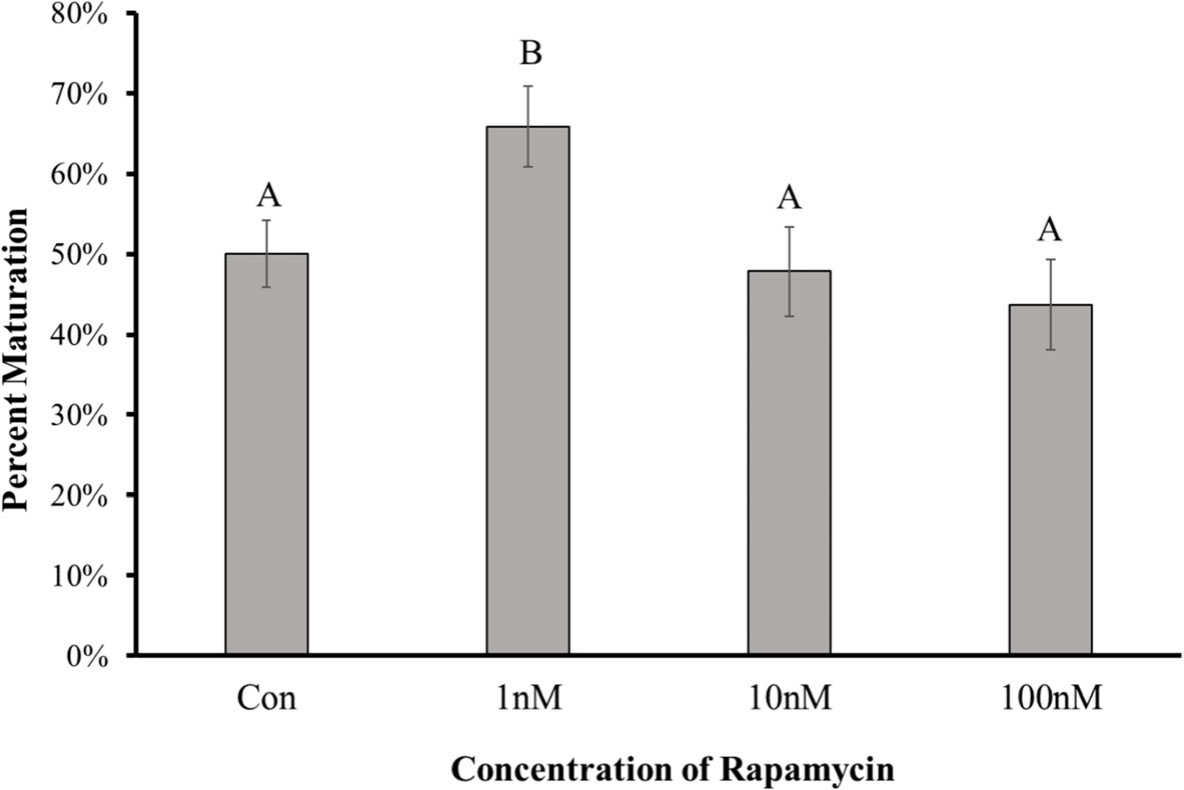
Low Concentration of Rapamycin Increases Maturation Rate. Oocytes underwent IVM with the HS/TN temperature treatment in the presence of vehicle control, 100 nM, 10 nM, or 1 nM rapamycin. The inclusion of 100 nM or 10 nM rapamycin in the IVM media had no effect on maturation rate after HS/TN IVM compared to vehicle control, though oocytes matured in the presence of 1 nM rapamycin had increased maturation rate compared to vehicle control

To determine if inducing autophagy in oocytes during HS increases the oocyte’s ability to mature to MII arrest, oocytes underwent IVM while experiencing HS during the first 21 h and TN conditions the following 21 h in the presence of vehicle control, 100 nM, 10 nM, or 1 nM rapamycin. Oocytes treated with all four concentrations of rapamycin were subjected to the HS/TN IVM treatment, based upon the effect of this temperature treatment on ATG12-ATG5 complex and LC3B-II abundance. The maturation rate of oocytes in the presence of DMSO vehicle control was 50.0 ± 4.1, 43.6 ± 5.6% for 100 nM rapamycin, 47.8 ± 5.5% for 10 nM rapamycin, and 65.8 ± 5.0% for 1 nM rapamycin (Fig. 5). Including 100 nM or 10 nM rapamycin in the IVM media had no effect on maturation rate after HS/TN compared to vehicle control (*P* = 0.31 and *P* = 0.72, respectively), though oocytes matured in the presence of 1 nM rapamycin had an approximately 1.3-fold increase in maturation rate compared to vehicle control (*P* = 0.03; Fig. 5). This data suggests low concentrations of rapamycin may provide the oocyte some resistance to HS and improve their ability to reach MII arrest, potentially through induction of autophagy.

## Discussion

Autophagy is the process by which cellular components are recycled and is activated by a variety of stressors [51]. In autophagy, the autophagosome forms around and sequesters damaged organelles or misfolded proteins, and proceeds to degrade its contents after interaction with a lysosome. BECN1 associates with PIK3C3 to increase nucleation of autophagosomes [19, 20], after which the autophagosome membrane is extended via two ubiquitin-like conjugation pathways including ATG12 or LC3. ATG12 forms a complex with ATG5 via an isopeptide bond, and this complex formation is indicative of increased autophagosome formation [21]. The cleavage of LC3-I to form LC3-II is indicative of autophagy occurrence, as cleavage of LC3-I and the conjugation of phosphatidylethanolamine allows for LC3-II to interact with the autophagosomal membrane [24, 52].

Whereas HS during IVM did not affect the abundance of BECN1 or BCL2L1 in MII oocytes, there was increased abundance of ATG12 in complex with ATG5 in MII oocytes after undergoing HS/TN IVM. This increase of ATG12-ATG5 complex formation suggests a rise in autophagosome formation [53], and this increase specifically as a result of HS during the first half of IVM could be due to HS being applied prior to transcriptional inactivation associated with germinal vesicle breakdown (GVBD). After GVBD, the oocyte is almost entirely transcriptionally quiescent [54], but oocytes that underwent the HS/TN temperature treatment during IVM experienced HS before GVBD at a time when a transcriptional response could have occurred.

We have previously characterized the blastocyst development rate of MII oocytes collected after either TN/HS or HS/TN IVM, used for in vitro fertilization (IVF). Decreased blastocyst development rate was observed in fertilized TN/HS oocytes compared to controls, yet blastocyst development rate from fertilized HS/TN oocytes did not differ from control treated oocytes [50]. Therefore, we used the HS/TN temperature treatment for IVM to further characterize autophagy in oocytes under HS conditions because this treatment decreases maturation rate, but a subset of oocytes can reach MII arrest and maintain the ability to produce blastocysts.

At 21 h of IVM, there was decreased abundance of LC3B-II in oocytes that had undergone HS compared to oocytes that underwent IVM in TN conditions, suggesting that HS exacerbates the decrease in LC3B-II. A decrease in LC3B-II abundance during IVM could be interpreted as an increase in autophagosome utilization, since at least in some cell types, LC3B-II is degraded when autophagosomes interact with the lysosome [25]. This inverse relationship between decreased LC3B-II abundance and increased ATG12-ATG5 complex formation could be explained by the fact that the ATG12-ATG5 complex detaches from the autophagosomal membrane after autophagosome formation is complete [55].

Autophagy and apoptosis are regulated in tight coordination, partly through BCL-2 family member proteins, which are important regulators of apoptosis during mammalian ovary development [26–30]. The dual role of BCL-2 family members to regulate both autophagy and apoptosis is mediated by the ability of BCL-2 and BCL2L1 to prevent apoptosis by inhibiting the formation of mitochondrial pores that release cytochrome C [31], or BCL-2 and BCL2L1 can interact with BECN1 to inhibit autophagy [32, 33].

We have previously detected an increase in BCL2L1 protein abundance in the pig ovary due to HS, and immunostaining of sectioned ovarian tissue detected increased BCL2L1 localization in prophase I-arrested oocytes and primary follicles of ovaries from post-pubertal gilts subjected to HS [43]. In this study the abundance of BCL2L1 in MII arrested oocytes after IVM was not affected by HS, suggesting increased BCL2L1 abundance in the oocyte is dependent on other biological contributors. The mechanisms regulated by BCL2L1 in an oocyte during maturation is potentially still affected by HS, as BCL2L1 regulates autophagy or apoptosis through protein-protein interactions [31, 32]. We therefore characterized colocalization of BCL2L1 with either BAX or BECN1 to determine if HS can affect BCL2L1 protein interactions.

Colocalization of BCL2L1 with BAX remained constant in oocytes regardless of time point of IVM or temperature treatment, although, based on relative fluorescence signal, there did appear to be a higher abundance of BAX in oocytes collected after 21 h of HS compared to TN oocytes. There was little to no colocalization of BECN1 with BCL2L1 in oocytes regardless of time point of IVM or temperature treatment, though HS appeared to induce more BECN1 protein. Based on the high degree of colocalization between BCL2L1 and BAX, as well as the fact this protein-protein interaction is well characterized in somatic cells [56], BCL2L1 may be inhibiting the release of cytochrome C in oocytes during IVM regardless of TN or HS conditions. The distinct lack of colocalization between BCL2L1 and BECN1 indicates that BCL2L1 is not inhibiting autophagy under the IVM conditions used in this study. This finding, coupled with the fact that HS affects abundance of autophagy-related proteins, suggests that HS is inducing autophagy in oocytes during maturation.

If autophagy has protective effects in terms of developmental competence on the oocyte, then artificially activating autophagy in oocytes undergoing HS should have a positive effect on meiotic maturation rate. Rapamycin induces autophagy in yeast [57] and mammalian cells [58], via the inhibition of mechanistic target of rapamycin (mTOR; formerly mammalian target of rapamycin) complex 1 (mTOC1). It is well characterized that mTOR is a central regulator of cellular metabolism and cell fate [59]. The mechanism of rapamycin inhibition of mTORC1 involves rapamycin forming a complex with FK506-binding protein (FKBP12), which binds directly to mTORC1 [60, 61]. While this mechanism is not completely understood, modeling of the rapamycin-FKBP12 complex bound to mTOR suggests rapamycin displaces the alignment of some mTORC1 substrates to the catalytic cleft [59, 62].

Autophagosome-like structures have been previously detected in oocytes and granulosa cells [42, 63, 64], and rapamycin inclusion in IVM media has beneficial effects in pig [65, 66] and bovine [67] oocytes. In this experiment, oocytes underwent IVM subjected to HS in the presence of rapamycin in the maturation media. The maturation rate of the oocytes in 10 or 100 nM rapamycin was not different from oocytes that matured in the presence of vehicle control, however, the maturation rate of oocytes in 1 nM rapamycin was increased compared to control. These results are similar to other experiments that characterize pig oocytes undergoing IVM in normal conditions in the presence of rapamycin [65]. Since mTOR is a major regulator of nutrient sensing and cell fate decision [59], there could be a threshold that exists where the negative effects of inhibition of mTORC1 outweigh the beneficial effects of autophagy induction. This threshold might explain why a low concentration of rapamycin increased the percentage of oocytes able to undergo IVM while higher concentrations did not. There could also be a threshold at which excessive induction of autophagy is detrimental to the oocyte, as autophagy and apoptosis regulation are tightly linked [56].

Embryonic development is dependent on oocyte competence, which is determined by the cytoplasmic contents of the oocyte [68, 69]. HS is associated with reduced oocyte developmental competence and embryonic development [7–9], and autophagy is a potential mechanism that the oocyte could utilize during HS to recycle damaged cellular components. Herein we demonstrate that HS affected autophagy-related proteins in maturing oocytes, and that pharmacological induction of autophagy increased oocyte maturation during HS.

## Conclusions

The results presented here suggest that HS increases autophagy induction in pig oocytes during IVM, and that artificial induction of autophagy, using 1 nM rapamycin, increases the maturation rate of oocytes during IVM. These data add to the understanding of the components of oocyte viability, which is necessary for improving the efficiency of assisted reproductive techniques and developing environmental stress mitigation strategies to improve reproduction [70, 71].

## Supplementary information

**Additional file 1: Supplementary Fig. 1**. Correlation predicting colocalization of BCL2L1 and interacting proteins. Oocytes that underwent either TN/TN IVM or HS/TN IVM were fixed and used for IHC to determine colocalization. The PDC colocalization plugin in the ImageJ processing program was used to calculate scatter plots representing colocalization of signal intensity collected from confocal microscopy of individual oocytes. Scatter plots representing colocalization of BCL2L1 (green) and BAX (red) signal suggest a high degree of colocalization based on overlapping signal (**A**). Scatter plots representing colocalization of BECN1 (green) and BCL2L1 (red) signal suggest little to no colocalization (**B**). **Supplementary Fig. 2**. Correlation comparison and relative fluorescence intensity. Oocytes that underwent either TN/TN or HS/TN IVM were fixed and used for IHC to determine colocalization (*n* = 3). The average Pearson’s correlation coefficient was compared between BCL2L1 and BAX fluorescence colocalization and BCL2L1 and BECN1 fluorescence colocalization for each treatment (**A**). The average relative fluorescence of BAX (**B**), BC2L1 (**C**), and BECN1 (**D**). Asterisks represents significant difference (*P* < 0.05) in the average correlation coefficient at each treatment per time point. Different superscripts denote significant difference (*P* < 0.05) between each treatment at each time point.

## Data Availability

Not applicable.

## Abbreviations

ATG: Autophagy related gene
BAX: BCL2 associated X, apoptosis regulator
BCL2L1: BCL2 like 1 protein
BECN1: Beclin1
COC: Cumulus-oocyte-complex
HS: Heat stress
IVM: In vitro maturation
GV: Germinal vesicle-intact
GVBD: Germinal vesicle breakdown
LC3B: Microtubule-associated protein 1 light chain 3 beta
mTOR: Mechanistic target of rapamycin
PVA: Polyvinyl alcohol
TN: Thermal neutral
