# Supplementary material for: Characterization of the effects of heat stress on autophagy induction in the pig oocyte
Source: Reprod Biol Endocrinol. 2021 Jul 9;19:107. doi: 10.1186/s12958-021-00791-4 (PMC8268447; doi:10.1186/s12958-021-00791-4)
Supplement: Supplementary file 1 — Additional file 1: Supplementary Fig. 1. Correlation predicting colocalization of BCL2L1 and interacting proteins. Oocytes that underwent either TN/TN IVM or HS/TN IVM were fixed and used for IHC to determine colocalization. The PDC colocalization plugin in the ImageJ processing program was used to calculate scatter plots representing colocalization of signal intensity collected from confocal microscopy of individual oocytes. Scatter plots representing colocalization of BCL2L1 (green) and BAX (red) signal suggest a high degree of colocalization based on overlapping signal (A). Scatter plots representing colocalization of BECN1 (green) and BCL2L1 (red) signal suggest little to no colocalization (B). Supplementary Fig. 2. Correlation comparison and relative fluorescence intensity. Oocytes that underwent either TN/TN or HS/TN IVM were fixed and used for IHC to determine colocalization (n = 3). The average Pearson’s correlation coefficient was compared between BCL2L1 and BAX fluorescence colocalization and BCL2L1 and BECN1 fluorescence colocalization for each treatment (A). The average relative fluorescence of BAX (B), BC2L1 (C), and BECN1 (D). Asterisks represents significant difference (P < 0.05) in the average correlation coefficient at each treatment per time point. Different superscripts denote significant difference (P < 0.05) between each treatment at each time point. [file 12958_2021_791_MOESM1_ESM.docx]

**Supplementary Figure 1. Correlation predicting colocalization of BCL2L1 and interacting proteins.**

**
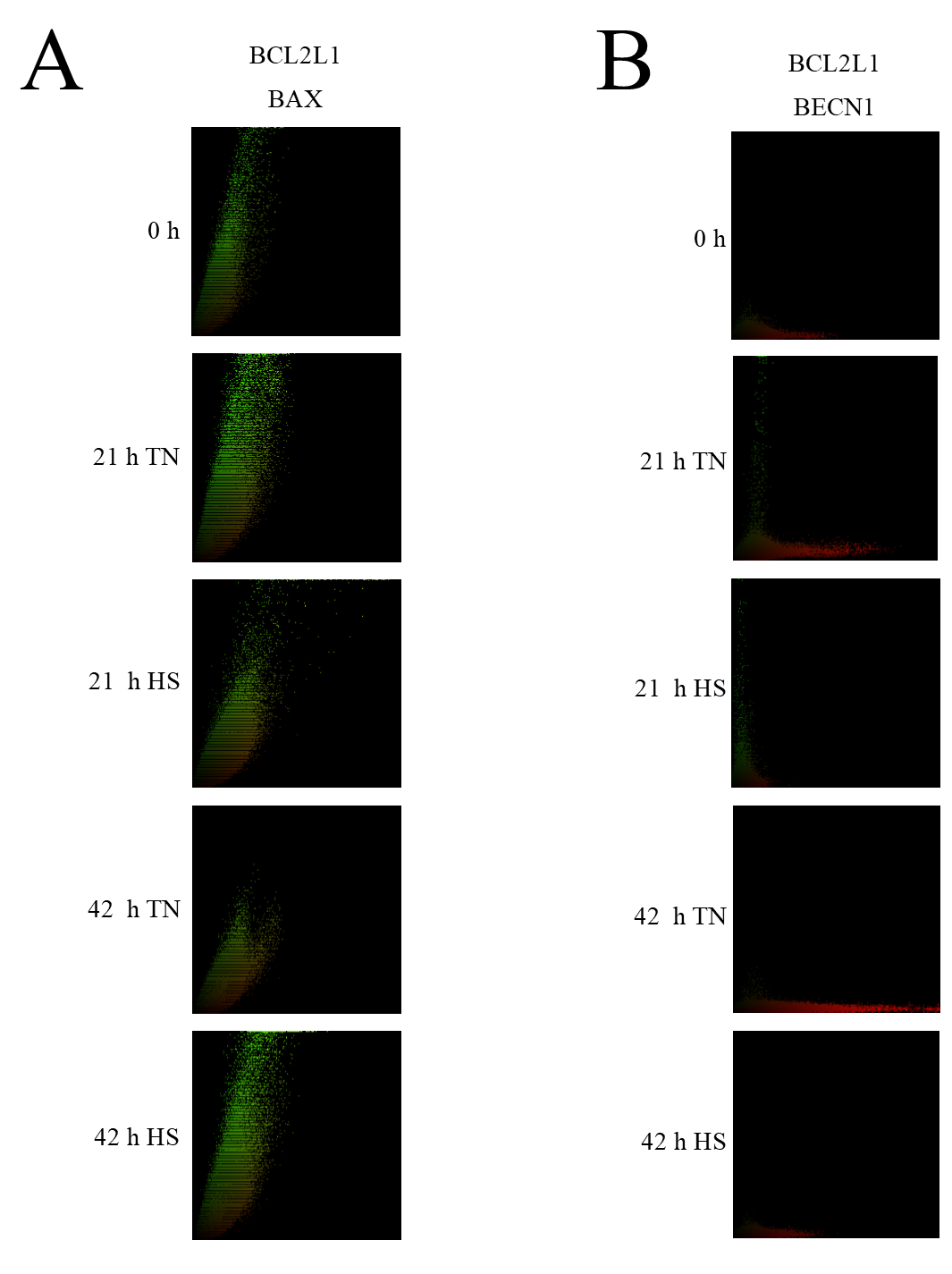
**

**Supplementary Figure 2. Correlation comparison and relative fluorescence intensity**

**
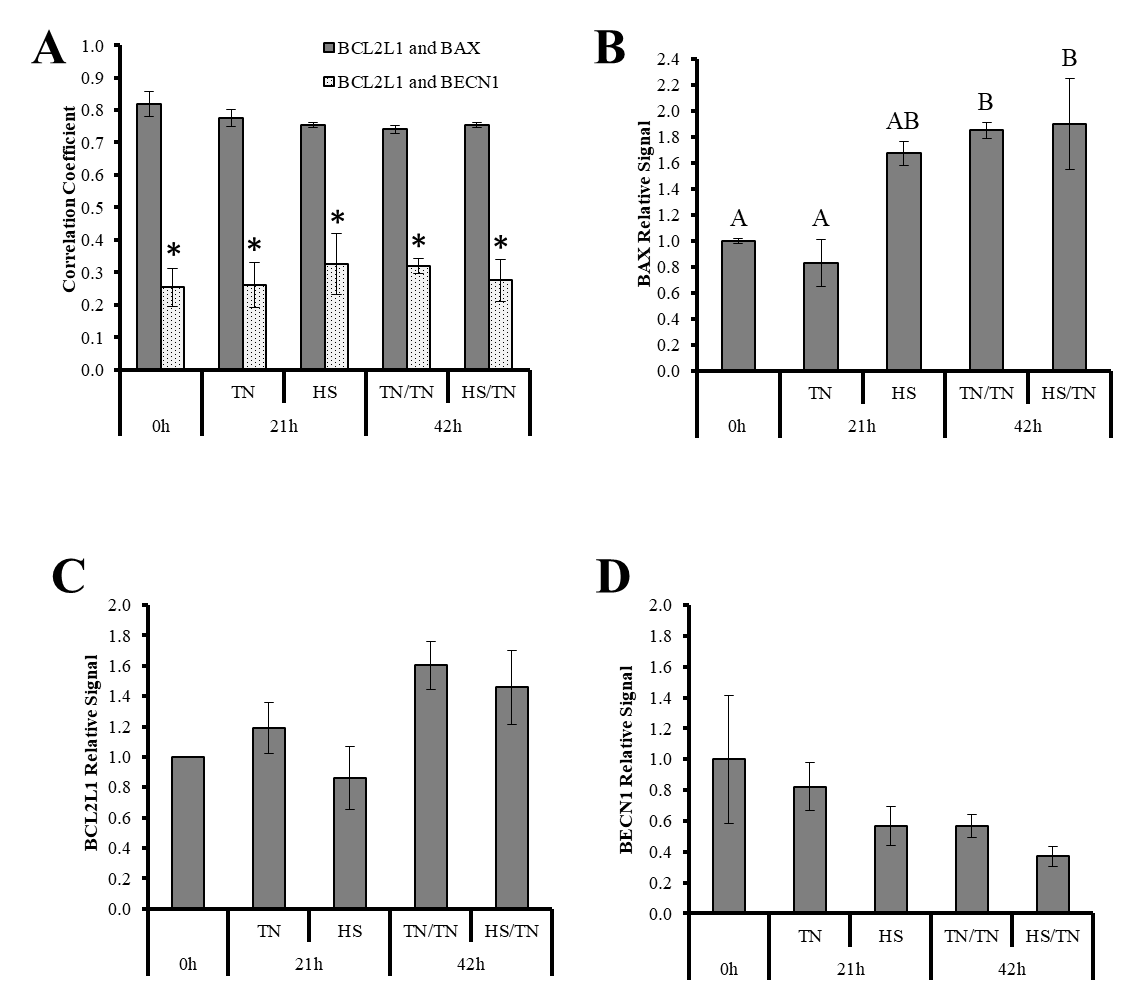
**
